# Treating infections with ionizing radiation: a historical perspective and emerging techniques

**DOI:** 10.1186/s13756-020-00775-w

**Published:** 2020-07-31

**Authors:** B. van Dijk, J. V. C. Lemans, R. M. Hoogendoorn, E. Dadachova, J. M. H. de Klerk, H. C. Vogely, H. Weinans, M. G. E. H. Lam, B. C. H. van der Wal

**Affiliations:** 1 Department of Orthopaedics, University Medical Center Utrecht, Utrecht, The Netherlands; 2 College of Pharmacy and Nutrition, University of Saskatchewan, Saskatoon, Canada; 3 Department of Nuclear Medicine, Meander Medical Center Amersfoort, Amersfoort, The Netherlands; 4 Department of Biomechanical engineering, TU Delft, Delft, The Netherlands; 5 Department of Radiology and Nuclear Medicine, University Medical Center Utrecht, Utrecht, The Netherlands

**Keywords:** Radioimmunotherapy, Radiotherapy, X-rays, Radiation, Infection, Biofilm, Orthopaedic infection, Periprosthetic joint infection, Inflammation, Anti-inflammation

## Abstract

**Background:**

Widespread use and misuse of antibiotics have led to a dramatic increase in the emergence of antibiotic resistant bacteria, while the discovery and development of new antibiotics is declining. This has made certain implant-associated infections such as periprosthetic joint infections, where a biofilm is formed, very difficult to treat. Alternative treatment modalities are needed to treat these types of infections in the future. One candidate that has been used extensively in the past, is the use of ionizing radiation. This review aims to provide a historical overview and future perspective of radiation therapy in infectious diseases with a focus on orthopedic infections.

**Methods:**

A systematic search strategy was designed to select studies that used radiation as treatment for bacterial or fungal infections. A total of 216 potentially relevant full-text publications were independently reviewed, of which 182 focused on external radiation and 34 on internal radiation. Due to the large number of studies, several topics were chosen. The main advantages, disadvantages, limitations, and implications of radiation treatment for infections were discussed.

**Results:**

In the pre-antibiotic era, high mortality rates were seen in different infections such as pneumonia, gas gangrene and otitis media. In some cases, external radiation therapy decreased the mortality significantly but long-term follow-up of the patients was often not performed so long term radiation effects, as well as potential increased risk of malignancies could not be investigated. Internal radiation using alpha and beta emitting radionuclides show great promise in treating fungal and bacterial infections when combined with selective targeting through antibodies, thus minimizing possible collateral damage to healthy tissue.

**Conclusion:**

The novel prospects of radiation treatment strategies against planktonic and biofilm-related microbial infections seem feasible and are worth investigating further. However, potential risks involving radiation treatment must be considered in each individual patient.

## Introduction

For more than a century, radiation has been used as a treatment modality for a wide range of diseases. Its usefulness in diagnosis and oncological treatment is undisputed, but in the early twentieth century, radiation was commonly employed to treat infections, especially due to a lack of alternative treatments and limited knowledge of possible side effects. In the 1940s, radiation treatment slowly became obsolete with the discovery and availability of antibiotics. However, the war against infections is still ongoing and widespread use and misuse of antibiotics have led to the emergence of antibiotic-resistant bacteria, while the discovery and development of new antibiotics is rapidly declining [1].

The field of orthopedic surgery is in dire need of novel treatments. Total joint replacements are a common, last-resort treatment for degenerative joint disease, but 1–4% of patients develop a periprosthetic joint infection (PJI) [2]. PJI is difficult to treat as bacteria form a biofilm on the prosthetic material. This hinders the host immune system, but more importantly, bacteria in a biofilm are mostly in a metabolic inactive or dormant state and therefore not susceptible to most antibiotics [3].

Currently, patients with PJI get prolonged antibiotic treatment, occasionally combined with multiple irrigation and debridement surgeries with- or without implant exchange to combat the infection. Despite this intensive treatment, outcomes are still unpredictable. In addition, the (often) elderly PJI population usually has multiple comorbidities, which necessitates multimodality treatment. In this regard, PJI patients are not dissimilar to oncology patients, with comparably high morbidity- and mortality rates. The 5-year mortality of PJI even surpasses that of most forms of prostate-, breast- and thyroid cancer [4, 5]. Interestingly, like in these previously mentioned oncological conditions, ionizing radiation may play a role in the treatment of infectious diseases.

Ionizing radiation therapies of the past, like x-ray- or radioactive iodine therapy, damaged a large area around the region of interest. However, recent advances in both external and internal radiation techniques make these therapies potentially more accurate. In external radiation treatment, these advances include intensity-modulated radiotherapy, as well as novel technologies like MR Linac [6]. Similarly, in internal radiation treatment, radioimmunotherapy (RIT) has allowed the delivery of cytotoxic radiation to specific target cells, through the coupling of antibodies and radioisotopes [7]. The same concept could be applied to treatment of infection, by coupling the radioisotopes to antibodies that targets bacterial cells or biofilm antigens [8]. With these advances, a re-evaluation of their merits in infection treatments seems warranted. This article therefore aims to provide a historical overview as well as future perspective of radiation therapy in infectious diseases with a focus on orthopedic infections.

## Methods

A systematic search strategy was designed for three academic databases, Pubmed, Embase and Cochrane, to select studies that used radiation for treatment of bacterial or fungal infections (Appendix 1). Studies were independently screened in two stages: screening of titles and abstracts, followed by the retrieval and screening of full-text publications. Two reviewers used predetermined inclusion criteria as described in Table 1. Conflicts were solved by consensus, or (if no consensus could be reached) through consultation of a third reviewer. Since most studies involving radiation treatment of infections were performed in the distant past, no restrictions were set on publication date. Reference screening and citation tracking of the included articles was performed. The included full-text publications were then divided into two main groups: studies investigating external radiation therapy and publications investigating internal radiation therapy. Since the included publications differed strongly in scope, disease and patient populations, results were clustered by their organ system or disease group.

**Table 1:** Eligibility Criteria

**External Radiation**	
**Inclusion Criteria**	**Exclusion Criteria**
Investigates treatment of bacterial or fungal infection with radiation	Diagnostic studies
Human, clinical study	Indirect use of radiation
	In vitro research
	No abstract/full-text available
	No English/German/Dutch language
**Internal Radiation**	
**Inclusion Criteria**	**Exclusion Criteria**
Investigates treatment of bacterial or fungal infection with radiation	Diagnostic studies
	No abstract/full-text available
	No English/German/Dutch language

## Results

**Fig. 1:**
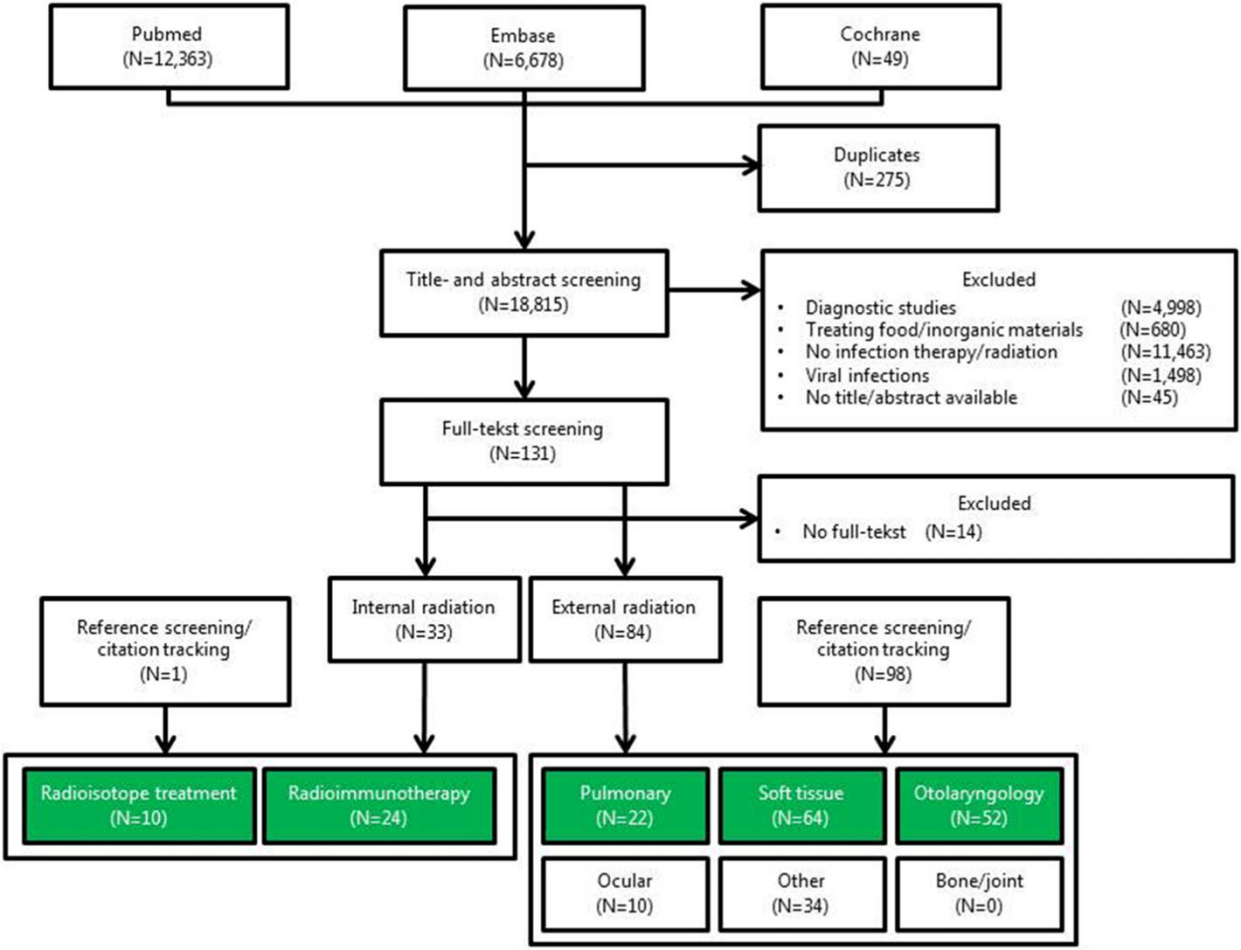
Flowchart of the systematic literature search

Of 18,815 studies, 216 potentially relevant full-text publications were reviewed and divided into two groups, external and internal radiation. In this review, external radiation is defined as a method for delivering a beam of x-rays to the infection site and internal radiation is defined as a systemic treatment, involving radioisotopes that deliver a cytotoxic level of radiation to an infected site. Through reference screening and citation tracking another 99 articles were found for a grand total of 216 articles in total (Fig. 1). Due to the large number of studies, different articles in different topics were chosen that can directly or indirectly correlate to orthopaedic infections. Unfortunately, there were no suitable articles for radiation therapy on PJI or osteomyelitis that could be included. The following topics were chosen and are described in detail below: For external radiation treatment, pneumonia, soft tissue infections, and otolaryngological infections were chosen. For internal radiation treatment, bone tuberculosis, *Helicobacter pylori* and RIT for bacteria and fungus were chosen.

### External radiation

#### Discovery of X-rays

In 1895, Wilhelm Röntgen was the first to describe the existence of X-rays [9]. Following the publication of a radiograph of his wife’s left hand, this new technique was welcomed with great enthusiasm. Already a few years later, the first therapeutic uses were described for infectious diseases.

#### Pneumonia treated with X-ray

Before the advent of antibiotics, pneumonia was a disease known for its high mortality [10]. Musser and Edsall, performing clinical experiments with x-rays, found that this radiation markedly improved condition and disease progress of leukemia patients, which they hypothesized was due to an increase in metabolic processes in tissues [10]. Unresolved pneumonia was, in their opinion, also a situation in which the body could not adequately metabolize the unresolved exudate that was left in the lungs. Based on this theory, they treated a patient who suffered from a 1 month old unresolved pneumonia with x-ray treatment for 5 min daily during 5 days. At the end of the week, the pneumonia had completely resolved [10]. Following this publication, multiple publications were published that also investigated the merits of x-rays in unresolved pneumonia, with good clinical results [11, 12]. Krost et al. then investigated x-ray treatment for pneumonia in 12 children with unresolved pneumonia [13]. These patients had symptoms for as long as 3–6 weeks before the first x-ray treatment was given. After 1–2 x-ray treatments, (5 mA, 5 min, spark gap 19 cm, distance 20 cm, 3 mm Al and 4 mm leather filter) 11 cases of pneumonia (92%) resolved within several days, the clinical situation often improved after hours. Powell et al. continued research of x-rays in the 1930’s, his cohort of adults showed a decreased mortality of 6.7% (9/134 patients), a sharp improvement from earlier mortality rates for pneumonia [14]. In that study, patients were alternatively included in the x-ray group or the control group, but after seeing the marked reduction in mortality in the x-ray treatment group, all control patients were subsequently treated with x-rays (all patients received 250–350 röntgen). A few years following Powell’s research, sulfonamides, the first antibiotics, were used as standard treatment for pneumonia, and use of x-rays fell out of favor. Research, however, was continued for patients who did not respond to, or did not tolerate sulfonamide therapy. In one such study, 22 out of 29 patients (75.9%) who showed no response to sulfonamides, recovered completely with x-ray therapy (120 Kv, distance 40 cm, 3 mm Al filter, 200 röntgen single-dose for a maximum of 3 doses) [15]. Some short-term adverse effects were shown by several authors, namely convulsions and cyanosis when the single session radiation dose exceeded 10 Gy [16, 17]. These complications often resolved, and therapy was still effective in these patients. Unfortunately, none of the authors performed long-term follow-up of their patients, so the long term radiation effects, as well as a potential increased risk of malignancies could not be investigated. For a comprehensive review of the clinical and animal literature on x-ray use in pneumonia, we direct the reader to the study by Calabrese and Dhawan [18].

#### Soft tissue infections treated with X-ray

Different soft tissue infections such as gas gangrene, furuncles and carbuncles were treated with X-rays in the first half of the twentieth century and will be discussed in detail below. Gas gangrene, or *Clostridium myonecrosis*, is a destructive soft-tissue infection caused by anaerobic *Clostridium* bacteria. The micro-organisms that are often associated with severe trauma or contaminated wounds thrive in low-oxygen environments and rapidly destroy muscle tissue while producing gas in the tissues. Severe pain, edema and/or bullae, an unusually rapid tachycardia, and palpable soft tissue crepitations are all clinical signs that point to the presence of gas gangrene [19]. Before the antibiotic era, surgery, namely amputation, was the only treatment, and mortality was around 50% [20]. Radiologist Kelly reported in 1931 his experience with treating gas gangrene with x-rays and found a mortality of only 2 in 8 patients, without the need for further amputation after x-ray treatment (6–7 doses of 3 min; spark gap 13 cm, 5 mA, distance 38 cm, 0.5 mm Al filter). He described this in his paper in one patient: “*The laboratory cultures were positive for Bacillus welchii, and x-rays films showed considerable gas in the soft tissues. Amputation was advised by consultants, but action was deferred to see the effects of the other treatment. Serum* [equine serum containing antibodies against one or more *Clostridium* species] *and x-ray therapy were administered. No amputation was necessary and the patient was dismissed after seven weeks hospitalization*” [21]. Following Kelly’s initial success, many studies were performed over the years, with the majority showing excellent results. In a review and meta-analysis of the case series literature, Kelly and Dowell showed that a combination of surgery, serum therapy and x-ray treatment (different radiation regimes were used during this study) resulted in a 11.5% mortality (42/364 patients) compared to a 35–50% mortality rate when only surgery and serum were evaluated together [20]. In a subgroup of x-ray patients who received multiple x-ray treatments, mortality was even lower, at 5.9% (17/288 in patients with ≥3 x-ray treatments). In a subgroup that underwent only x-ray treatment without serum therapy, mortality was 4.7% (2/42 patients) and no amputations were necessary. How x-rays halted the gas gangrene infection was never elucidated, although it was generally known that the relatively low radiation dose was not able to destroy the bacteria directly. More likely hypotheses that were proposed included the possibility that radiation causes local vessels to dilate, increasing oxygen supply to the infected tissue and thus diminishing the potency of anaerobic bacteria, as well as the possibility that radiation stimulated either the proliferation of immune cells or the release of bactericidal products from lymphocytes [22, 23]. It must be noted that some authors did not find x-rays to be effective [24], and that the promising mortality figures could have been the result of selection bias as well as an improved standard of care for these infections over time [25].

A furuncle, or boil is an infection of the hair follicle and its surrounding tissue caused by *Staphylococcus aureus* or *Staphylococcus epidermidis* which are also the most common pathogens causing PJI today. When multiple furuncles fuse together it is called a carbuncle, both had high mortality rates in the first half of the twentieth century, before the use of antibiotics. As early as 1906, Coyle described complete abortion of the carbuncle in 4/5 patients treated with x-rays [26]. This result wasn’t given much attention until almost a decade later, when Dunham published the results of 67 patients that were treated with a single x-ray dose of 6 Gy and stated that “*nothing in all roentgen therapy gives such positive and uniformly perfect results as the treatment of a carbuncle*” [27]. In the following years, multiple articles were published about the great and prompt benefit to patients treated with x-rays [28]. A lower single therapeutic dose of 0.75–2 Gy showed less radiation-induced side effects and an even greater effect on pain reduction and healing, especially in early stages of the disease [29]. In the early 1940’s, this x-ray therapy became obsolete due to the introduction of antibiotics. For a more detailed description of the historical role of x-ray treatment for carbuncles and furuncles we direct the reader to the review by Calabrese [29].

#### Otolaryngological applications

Before the advent of tympanostomy tubes, otitis media was a major health problem in school children. Following upper respiratory tract infections, tissue in the nasopharynx swells and blocks the Eustachian tube, thus blocking the outflow of middle ear secretions, which may become infected and cause conductive hearing loss. Blockage of the Eustachian tube may also be caused by swelling of the adenoid tissue of the nasopharynx [30]. Treatment in the past consisted of paracentesis, adenoidectomy or surgical removal of tissue surrounding the Eustachian tubes, although these therapies were often ineffective [31]. The resulting chronic hearing loss had a deleterious effect on the development of normal hearing and speech of children.

Early in the twentieth century, x-rays were proposed as a viable treatment to otitis media caused by Eustachian tubes blocked by lymphoid tissue, as it was already known that these tissues were very radiosensitive [32]. Beattie et al. found in 1920 that patients suffering from chronic otitis media with symptoms of mastoiditis showed clinical improvement after diagnostic mastoid x-rays. Out of 14 chronic patients, 9 improved after only 1–3 sessions with 180 s of x-ray exposure [33]. Similar results were found by other studies over the years [34].

Crowe and Baylor, happy with the effect that radiation had in reducing lymphoid tissue around the Eustachian tube, proposed that radiation could be applied much more locally compared to x-ray through nasal application of a small radioactive radium or radon source, which would cause much less systemic radiation [35]. Through covering the applicator with brass, all alpha- and most beta-radiation was filtered. Gamma rays were emitted that mimicked the x-ray treatment, but applied only locally, where it was needed. The technique was optimized by Crowe and colleagues, and a nickel-copper alloy was used instead of brass to cover the applicator, so that more beta-radiation was emitted that decreased the necessary application time and reduced the gamma-radiation load on tissues other than the nasopharyngeal lymphoid tissue. The treatment differed between studies but often consisted of 1–4 sessions of application with around 25–50 mg ^226^Ra sulphate for 8–15 min (~ 5 Sv at lymphoid tissue over 6 sessions, total dose in surrounding tissues estimated to be 36–142 Sv) [36–38]. The efficacy of the treatment was excellent, symptoms decreased within days, and the radium treatment was used in many children, but also in thousands of air force pilots and submarine personnel who had undergone baro-trauma [39].

The positive results in children were illustrated in a randomized controlled trial by Hardy and Bordley, which consisted of over 1000 school children with conductive hearing loss who were randomized in groups that received three sessions with an applicator containing either radium or a placebo, blinded to patient and physician [40]. In the subgroup with greatest hearing loss (i.e. the group with large lymphoid tissue overgrowth), hearing improved significantly greater with radium therapy compared to control treatment, and lymphoid tissue was significantly reduced. Interestingly, mild hearing loss in the placebo group improved markedly over the years as well, from which it was concluded that radium therapy should only be performed in cases in which hearing loss is found as a result of Eustachian tube dysfunction, because in most other cases, the condition also improved without treatment.

Over time, physicians became more concerned about the potential long-term health effects. An increase in cancer risk was suggested by some studies that followed children who had received radiation for benign conditions during childhood [41, 42]. However, these increased cancer risks were never unequivocally shown in cohort studies that investigated patients treated with nasopharyngeal radium. A cohort by Ronckers et al. found no increase in head and neck- or thyroid malignancies in a large cohort of over 4000 patients, although the incidence of breast cancer and non-Hodgkin lymphoma was slightly elevated [38]. Another study by Yeh et al. found no significant increase in the incidence of malignancies in a cohort of more than 1700 patients with around 40 years of follow-up [43]. Loeb et al. performed a literature review of studies on nasal radium therapy that included almost 30,000 patients (of whom a large proportion was treated by Crowe and colleagues). They found no cases of malignancies that could be clearly attributed to radium treatment [44].

Although an increased incidence of malignancies was never proven, the use of radium was not without risks. Notable was an incident in 1958 at the otolaryngology department of our own institution, the University Medical Center Utrecht, where the tip from a radium capsule broke away from the applicator, and was accidentally swallowed, with the treating physician being unaware. The 5-year old patient returned home, where she threw up the capsule, which was then accidentally deposited into the chimney by her father. The charred (and radioactive) ashes were distributed outside, thus contaminating the entire house and garden with radioactive material. This prompted a citywide emergency, the patient and her family were quarantined, and all persons who had contacted the family during the incident had to be examined both medically, and with Geiger counters (Video 1). During the first month after the incident, parts of the house were broken down and renovated by army personnel in protective gear. The radioactive waste was dumped in the ocean, some 30 miles from the Dutch coast. A few months after the incident, a new “Radioactive substance decree” was written into Dutch law, detailing “( …) *that sources of Radium could only exceed 1 mCu if, and only if, adequately encapsulated by a shell that cannot be removed without damage* ( …), *which is hermetically sealed and which is created from an indestructible material* ( …)” [31]. Unfortunately, this measure came too late. The incident caused much media publicity, and with increasing fear of radioactive substances, fueled more so by the Cold War, radium therapy was quickly abandoned in the Netherlands, also partly because of the advent of non-radioactive alternatives. An in-depth description of this incident was written by Graamans [45]. The patient was said to have lived a healthy life, with no radiation-related complications.

**Video 1: The follow-up of the radium applicator incident in the Dutch city of Putten**. [Transcript: The small town of Putten in the area called “De Veluwe” has gained worldwide attention, because the house of the Haanschoten family in the “Schoolstraat” was contaminated with radioactive material after a medical treatment. Quickly following the discovery, the immediate surroundings of the house were isolated. The garden, in which radioactive ashes was scattered, was covered with a plastic tarp, to prevent contaminated dust being blown away by the wind. All inhabitants of the town that had been in contact with the Haanschoten family were medically examined. They had to go to the police headquarters in Putten. At the police station they were investigated with devices that could detect the presence of radioactivity. Luckily, nobody was found to be contaminated during the investigation. Among them, the friend of the 5 year old Joke Haanschoten, who had to leave her house in Putten and who had to be admitted and observed at the hospital in Utrecht, together with her parents, brother and sisters.]. **Source: Dutch Institute for Image and Sound,****https://eye.openbeelden.nl/media/665796**. **No alterations to original work, CC BY-SA 3.0 NL.**

### Internal radiation

In this review, internal radiation is defined as a systemic treatment, involving radioisotopes that deliver a cytotoxic level of radiation to a diseased site. The hypothesis of “magic bullets” that could selectively kill pathogens or cells without harming healthy tissue was first described around 1900 by Paul Ehrlich [46]. The concept of targeted radiation therapy was used from the 1900s for different infectious diseases and is described in detail below.

#### Thorium X

Starting from around 1912, Thorium X was used in dermatology and as a treatment for rheumatic diseases. Thorium X (Radium-224; ^224^Ra) is a short-lived alpha-emitter (half-life of 3.6 days) and was applied topically, intravenously and orally. Around 1940, Peteosthor was developed to treat bone tuberculosis [47]. The drug contained ^224^Ra-chloride (Thorium X), platinum and red dye eosin. The hypothesis was that this short-lived bone-seeking alpha-emitter could selectively target, accumulate, and destroy the infected bone. Between the 1940’s, and mid-1950’s, primarily children and juveniles were treated with high doses of ^224^Ra, receiving repeated injections up to 2 MBq twice a week, often for prolonged periods of time, sometimes totaling up to 140 MBq [48]. Around 1950, Spiess and Mays questioned the efficacy of Peteosthor and conducted several in vitro and in vivo experiments. They showed that killing of *Mycobacterium tuberculosis* was seen in vitro with high doses of ^224^Ra, but no killing was seen in vivo. Objections to the treatment were raised in the early 1950’s, the primary one being that ^224^Ra deposited in the growing skeleton of children and juveniles would cause severe damage [48]. Because of the questionable efficacy of the treatment and the introduction of antibiotics like Streptomycin, discovered by Waksman (1943), Peteosthor was abandoned as a treatment for bone tuberculosis in 1956. After 1956, Spiess and Mays followed a cohort of 899 patients treated with high doses of Peteosthor for many years. A significant increase was seen in the incidence of bone tumors (56 cases among 899 patients, 6.2%) [47].

#### Iodine-131 – helicobacter pylori

*Helicobacter pylori* (Hp) infection is a common chronic bacterial infection, present in almost half of the world population [49]. Multiple studies investigated the effect of radioactive iodine-131 (^131^I) on Hp. ^131^I is a short-lived beta-emitter (half-life 8.4 days) and is an important treatment modality in the management of thyroid cancer and hyperthyroidism. ^131^I does not only accumulate in the thyroid, but also in the stomach, and could therefore potentially eradicate Hp infection [50]. In 71 patients treated for differentiated thyroid carcinoma, a pre-treatment urease breath test was done to diagnose an Hp infection. Twenty-three patients had a negative post-treatment result and thus a significant reduction in Hp. [50] In another study, 18 of 85 patients infected with Hp who were treated for hyperthyroidism with ^131^I showed a negative urease breath test after treatment, which also means a significant reduction in Hp. [51] However, no significant reduction was seen in two other studies, the first with 18 patients treated for differentiated thyroid carcinoma and the second study with 76 patients treated for differentiated thyroid cancer and 11 for primary hyperthyroidism [52, 53].

#### Radioimmunotherapy

Currently, RIT is used to treat different types of cancer, but until the 1940’s, cancer treatment was exclusively based around the surgical approach. That changed with the advent of molecular medicine, and with the discovery of “chemotherapy” by Louis Goodman and Alfred Gilman [54]. In the next few decades, multiple chemotherapeutic agents were discovered that successfully induced remission of multiple types of cancer. However, during the development of these systemic cancer drugs, significant problems, such as acute and long-term toxicities were repeatedly encountered. Therefore, a change of strategy was needed and was found in targeted-therapy [54]. The aim of targeted therapy is to specifically target tumor cells with specific antibodies or small molecules that interfere with molecular pathways related to carcinogenesis and tumor growth. In the late 1980’s, researchers shifted their focus to unraveling and understanding these molecular pathways and due to innovations in technology more and more antibodies and inhibitors of specific targets were discovered [55]. While antibodies can directly affect tumor cells, they can also be used as transport vehicles to deliver agents that can destroy tumor cells (e.g. radioisotopes) [17]. When antibodies are labeled with radioisotopes, a high dose of ionizing radiation can be delivered directly to the targeted cells. In the past decade, success was seen in treating non-Hodgkin lymphoma with the only two radioimmunoconjugates approved by the FDA, ^131^I-tositumomab and ^90^Y-ibritumomab tiuxetan [7, 56].

#### Radioimmunotherapy of fungal infections

In vitro experiments showed that both planktonic cells and biofilms of *Cryptococcus neoformans* (CN) are susceptible to RIT. In vitro, CN-specific monoclonal antibodies conjugated to bismuth-213 (^213^Bi; short-lived alpha-emitter, half-life 45 min.) caused a 50% reduction in metabolic activity of the fungal biofilm and a 70% reduction in metabolic activity of planktonic cells at a dose of 1.11 MBq (30 μCi) when compared to the control non-specific antibody conjugation [57]. In the same study, 14.8 MBq (400 μCi) rhenium-188 (^188^Re; short-lived beta-emitter, half-life 17 h.) conjugated to CN-specific antibodies showed a reduction in metabolic activity of planktonic cells of 83%, but no reduction was seen in the metabolic activity of the biofilm [19].

In an in vivo experiment by Dadachova et al., nine groups of 10 mice were infected with 10^5^ CN cells. Multiple treatment groups were treated with intravenously administered specific antibodies bound to ^213^Bi and ^188^Re, a dose of 3.7 MBq (100 μCi) RIT showed a survival of 60% with ^213^Bi and 40% with ^188^Re on day 75 post-therapy when compared to 0% survival in the ‘cold’ antibody conjugates (antibodies without radioconjugates) and a saline-treated group [58]. In another study with the same in vivo CN model, RIT with ^213^Bi was compared to the antimycotic drug amphotericin. RIT was more effective in reducing fungal burden in lungs and brains, measured by colony forming unit (CFU) count in post mortem organs, where ^213^Bi conjugates could completely clear the infection, while amphotericin could not reduce that number of fungal cells [59].

#### Radioimmunotherapy of bacterial infections

Dadachova et al. also used RIT to combat bacterial infections. In vitro tests with ^213^Bi radiolabeled antibodies against *Streptococcus pneumoniae* showed minimal but significant killing when doses of 0.11–0.15 MBq (3–4 μCi) were used [60]. A higher dose could potentially have a higher bactericidal effect. Two in vivo experiments were done with C57BL/6 mice infected intra-peritoneally with 1000 CFU of *Streptococcus pneumonia.* In the first experiment, mice were treated with either ^213^Bi specific antibodies or “cold” antibodies, one group was left untreated. After 14 days, 87% of the mice treated with ^213^Bi survived versus 40% in the other two groups. In the second in vivo study, the mice were treated with 2.96 MBq (80 μCi) ^213^Bi labeled specific and non-specific antibodies. Unlabeled antibodies and an untreated group were used as controls. Mice treated with ^213^Bi labeled specific antibodies showed a 100% survival after 14 days versus 20% in the Bi^213^ bound non-specific antibody group and 60% in the unlabeled antibody and untreated group [60].

In another study, RIT with ^213^Bi showed prolonged survival in mice infected with *Bacillus anthracis* bacterial cells compared to control groups with unlabeled antibodies and phosphate-buffered saline (PBS) [61]. These results showed the therapeutic potential of RIT on infectious diseases [8]. Until now, there is no literature on using RIT to treat infections in humans.

## Discussion

Throughout history, humanity has battled infections and the war is still going on today. With an increasing incidence of antimicrobial-resistant bacteria, finding effective treatments has become increasingly important. In the last century, different treatments have been developed and later abandoned. However, with new techniques, and the need to move away from our dependency to antibiotics, it is not unwise to give older strategies renewed consideration. Also, gathered knowledge on therapies from other fields in healthcare could potentially be used to treat infections. This review aimed to provide a summary of both historical and recent advances in radiation treatment for infections, whilst providing insight in how to proceed forward and learn from mistakes made in the past. Both external and internal radiation have the potential to clear infections as shown in this review. However, collateral damage to healthy tissue is a major concern, especially in external radiation treatment. To treat infections with external gamma-radiation, a high dose is needed to kill the bacteria. As a consequence, the long-term risk of cancer increases in patients who are exposed to these high doses of radiation. Of course, X-ray therapy for infections largely preceded the onset of advances in linear particle accelerators and radiotherapy; therefore, radiotherapy has mostly been ignored as a potential candidate in infection treatment, especially since antibiotics were highly effective and widely available. As we are entering an era in which antibiotics are increasingly failing, a renaissance of external radiation therapy of infections may develop with stereotactic radiation therapy, intensity-modulated radiation therapy and MR guided radiotherapy becoming potential last resort treatments for resistant infections.

**Fig. 2:**
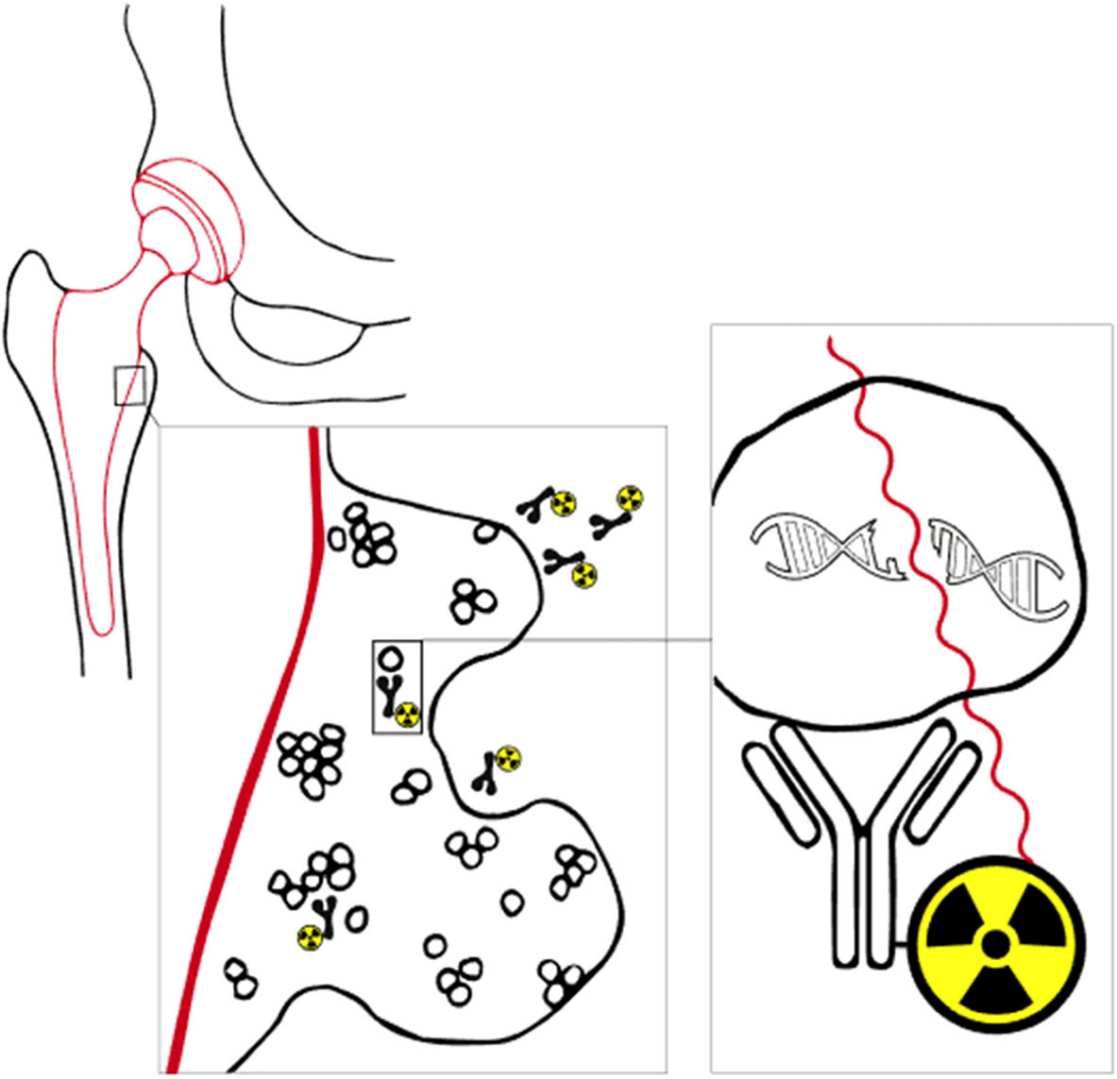
Concept: Radioimmunotherapy for periprosthetic join infections. Bacteria form a biofilm on the hip prosthesis that protects them from antibiotics and the immune system. Targeted radiation therapy with alpha- or beta-emitting radioisotopes could be able to destroy the structure of the biofilm and kill the bacteria

In contrary to these therapeutic techniques based on gamma radiation, alpha- and beta emitting radioisotopes can also be used for infection treatment. These radioisotopes have less penetrating power but are much more destructive, especially alpha-radiation. As early as 1950, the bactericidal effect of alpha-emitting radioisotope ^224^Ra was shown in vitro [62]. This makes them particularly interesting to use as Paul Ehrlich’s “Magic bullets” that can target bacteria or the biofilm, while minimizing collateral damage to healthy tissue. Key in internal radiation treatment for infections is to bring the radioisotopes in close vicinity to the target. For example, ^224^Ra has bone-seeking properties as it is a calcimimetic and is therefore incorporated into bone with increased bone-turnover such as bone infections. However, in subsequent clinical studies where ^224^Ra is used to treat bone tuberculosis, even extremely high doses were not effective and over time, led to a significant increase of bone tumors [47]. This suggests that a more selective targeting is necessary to utilize the full potential of these alpha- and beta-emitting radionuclides. Dadachova et al. showed that using antibodies as a transport vehicle for delivery of radioisotopes, bacteria and fungi can be targeted with high specificity, comparable to how RIT is used in the field of oncology. RIT relies on the antigen-binding characteristics of the antibodies to deliver cytotoxic radiation to target cells. As microbes express antigens that are unique and different from host antigens, they can be targeted with high specificity and low cross-reactivity. It could especially be of great value in biofilm-related infections where dormant cells are metabolic inactive and therefore not susceptible to most antibiotics because the damaging effects of radiation are independent of the cell’s metabolic state. To improve RIT further, smaller vehicles can be used such as nanobodies. These nanobodies are derived from camelids and are ten times smaller than conventional antibodies. Due to their size, nanobodies have increased elimination to get rid of the potential dangerous remaining unbound radioimmunoconjugates minimizing collateral damage even further. Also, they have considerable better penetration into tissue and presumably the biofilm [63]. Other advantages include high stability, solubility, expression, and specificity. Theoretically, a patient with a PJI where the hip implant is colonized with bacteria and a biofilm, could be treated with nanobodies labeled with an alpha-emitter like ^213^Bi or ^225^Ac that can penetrate deep in the biofilm, destroy the architecture and kill bacteria. (Fig. 2) These antibodies could also be a powerful diagnostic tool for positron emission tomography (PET)-imaging when labeled with positron-emitting radioisotopes such as fluorine-18 (^18^F) or zirconium-89 (^89^Zr). Due to the high specificity and rapid clearance, low background signal is expected so that even low-grade infections could be detected with high specificity and sensitivity.

Treatment and diagnostics with radiation is always prone to safety concerns. Alpha- and beta-emitting radioisotopes such as ^223^Ra and ^188^Re are already used in the clinic for metastatic castration-resistant prostate cancer. Safety studies show that treatment with these radioisotopes is associated with minimal adverse events [64, 65]. Nonetheless, it is important to consider survival time, age, physical and emotional wellbeing and alternative treatment options. As the 5-year survival of PJI patients is lower than the predicted survival for melanoma, prostate and breast cancer, aggressive treatments seem justified. Sometimes, infection surgery yields great risk to the point that only lifetime antibiotics or amputation is an option. Further development of antibiotic resistance due to antibiotic treatment reduces the chance of successful treatment even further. In these cases radiation treatment could be beneficial despite the possible long-term effects although these risks may be limited.

## Conclusion

The need for alternative treatment options for patients with (implant) infections like PJIs grows every year, not only due to increasing pathogen resistance to antibiotics, but also because biofilm formation obstructs the treatment of these infections with antibiotics. The novel prospects of radiation treatment strategies against planktonic and biofilm-related microbial infections are worth investigating further.

## Data Availability

Not applicable.

## Appendix 1. Pubmed, Embase and Cochrane search

Pubmed: ((((((Infection [Mesh] OR Infection [tiab] OR Infections [tiab] OR Infective [tiab] OR infectious [tiab])))) OR (((“Bacteria”[Mesh] OR Bacteria [tiab] OR Bacterial [tiab] OR Bacterium [tiab] OR Fungus [tiab] OR Fungi*[tiab] OR Fungal [tiab] OR Yeast [tiab] OR Yeasts [tiab]))))) AND (((((((“Radioisotopes”[MeSH] OR radionuclide [tiab] OR radionuclides [tiab] OR “Radioactive Isotope”[tiab] OR “Radioactive Isotopes”[tiab] OR radioisotope [tiab] OR radioisotopes [tiab] OR Radiation, Ionizing [MeSH] OR “ionizing radiation”[tiab] OR “alpha ray”[tiab] OR “alpha rays”[tiab] OR “alpha radiation”[tiab] OR “alpha particle”[tiab] OR “alpha particles”[tiab] OR “beta ray”[tiab] OR “beta rays”[tiab] OR “beta particle”[tiab] OR “beta particles”[tiab] OR “beta radiation”[tiab] OR “gamma ray”[tiab] OR “gamma rays”[tiab] OR “gamma radiation”[tiab] OR “roentgen”[tiab] OR “rontgen”[tiab] OR “Elements, Radioactive”[MeSH] OR “radioactive element”[tiab] OR “radioactive elements”[tiab] OR Radiolabel*[tiab])))) AND (((“Therapeutics”[Mesh] OR therapeutic [tiab] OR therapeutics [tiab] OR therapy [tiab] OR therapies [tiab] OR treatment [tiab] OR treatments [tiab]))))) OR (((Radioimmunotherapy [MeSH] OR radioimmunotherap*[tiab] OR immunoradiotherap*[tiab] OR RIT [tiab])))).

Embase: (((‘infection’/exp. OR Infection:ab,ti OR Infections:ab,ti OR Infective:ab,ti OR infectious:ab,ti) OR (‘bacterium’/exp. OR bacteria:ab,ti OR bacterial:ab,ti OR bacterium:ab,ti OR fungus:ab,ti OR fungi*:ab,ti OR fungal:ab,ti OR yeast:ab,ti OR yeasts:ab,ti)) AND (((‘radioisotope’/exp. OR radionuclide:ab,ti OR radionuclides:ab,ti OR ‘radioactive isotope’:ab,ti OR ‘radioactive isotopes’:ab,ti OR radioisotope:ab,ti OR radioisotopes:ab,ti OR ‘particle radiation’/exp. OR ‘ionizing radiation’:ab,ti OR ‘alpha ray’:ab,ti OR ‘alpha rays’:ab,ti OR ‘alpha radiation’:ab,ti OR ‘alpha particle’:ab,ti OR ‘alpha particles’:ab,ti OR ‘beta ray’:ab,ti OR ‘beta rays’:ab,ti OR ‘beta particle’:ab,ti OR ‘beta particles’:ab,ti OR ‘beta radiation’:ab,ti OR ‘gamma ray’:ab,ti OR ‘gamma rays’:ab,ti OR ‘gamma radiation’:ab,ti OR ‘roentgen’:ab,ti OR ‘rontgen’:ab,ti OR ‘radioactive element’/exp. OR ‘radioactive element’:ab,ti OR ‘radioactive elements’:ab,ti OR radiolabel*:ab,ti) AND (‘therapy’/exp. OR therapeutic:ab,ti OR therapeutics:ab,ti OR therapy:ab,ti OR therapies:ab,ti OR treatment:ab,ti Or treatments:ab,ti)) OR (‘radioimmunotherapy’/exp. OR radioimmunotherap*:ab,ti OR immunoradiotherap*:ab,ti OR RIT:ab,ti))) AND [embase]/lim NOT [medline]/lim.

Cochrane: ((Infection:ab,ti OR Infections:ab,ti OR Infective:ab,ti OR infectious:ab,t) OR (Bacteria:ab,ti OR Bacterial:ab,ti OR Bacterium:ab,ti OR Fungus:ab,ti OR Fungi*:ab,ti OR Fungal:ab,ti OR Yeast:ab,ti OR Yeasts:ab,ti)) AND (radionuclide:ab,ti OR radionuclides:ab,ti OR Radioactive Isotope:ab,ti OR “Radioactive Isotopes”:ab,ti OR radioisotope:ab,ti OR radioisotopes:ab,ti OR “ionizing radiation”:ab,ti OR “alpha ray”:ab,ti OR “alpha rays”:ab,ti OR “alpha radiation”:ab,ti OR “alpha particle”:ab,ti OR “alpha particles”:ab,ti OR “beta ray”:ab,ti OR “beta rays”:ab,ti OR “beta particle”:ab,ti OR “beta particles”:ab,ti OR “beta radiation”:ab,ti OR “gamma ray”:ab,ti OR “gamma rays”:ab,ti OR “gamma radiation”:ab,ti OR “roentgen”:ab,ti OR “rontgen”:ab,ti OR “radioactive element”:ab,ti OR “radioactive elements”:ab,ti OR Radiolabel*:ab,ti) AND ((therapeutic:ab,ti OR therapeutics:ab,ti OR therapy:ab,ti OR therapies:ab,ti OR treatment:ab,ti OR treatments:ab,ti) OR (radioimmunotherap*:ab,ti OR immunoradiotherap*:ab,ti OR RIT:ab,ti)).
